# Co-Delivery System of Curcumin and Colchicine Using Functionalized Mesoporous Silica Nanoparticles Promotes Anticancer and Apoptosis Effects

**DOI:** 10.3390/pharmaceutics14122770

**Published:** 2022-12-11

**Authors:** Khaled AbouAitah, Ahmed A. F. Soliman, Anna Swiderska-Sroda, Amr Nassrallah, Julita Smalc-Koziorowska, Stanislaw Gierlotka, Witold Lojkowski

**Affiliations:** 1 Medicinal and Aromatic Plants Research Department, Pharmaceutical and Drug Industries Research Institute, National Research Centre (NRC), 33 El-Behouth Street, Dokki, Giza 12622, Egypt; 2 Drug Bioassay-Cell Culture Laboratory, Pharmacognosy Department, Pharmaceutical and Drug Industries Research Institute, National Research Centre (NRC), 33 El-Behouth St, Dokki, Giza 12622, Egypt; 3 Laboratory of Nanostructures and Nanomedicine, Institute of High Pressure Physics, Polish Academy of Sciences, Sokolowska 29/37, 01-142 Warsaw, Poland; 4 Biochemistry Department, Faculty of Agriculture, Cairo University, Giza 12613, Egypt; 5 Laboratory of Semiconductor Characterization, Institute of High Pressure Physics, Polish Academy of Sciences, Sokolowska 29/37, 01-142 Warsaw, Poland

**Keywords:** co-delivery system, natural agent, cancer cell, curcumin combined colchicine core-shell nanoformulation, functionalized mesoporous silica nanoparticle, active cancer targeting

## Abstract

Purpose: Many natural agents have a high anticancer potential, and their combination may be advantageous for improved anticancer effects. Such agents, however, often are not water soluble and do not efficiently target cancer cells, and the kinetics of their action is poorly controlled. One way to overcome these barriers is to combine natural agents with nanoparticles. Our aim in the current study was to fabricate an anticancer nanoformulation for co-delivery of two natural agents, curcumin (CR) and colchicine (CL), with a core-shell structure. Using cancer cell lines, we compared the anticancer efficacy between the combination and a nanoformulation with CL alone. Methods: For the single-drug nanoformulation, we used phosphonate groups to functionalize mesoporous silica nanoparticles (MSNs) and loaded the MSNs with CL. Additional loading of this nanoformulation with CR achieved the co-delivery format. To create the structure with a core shell, we selected a chitosan–cellulose mixture conjugated with targeting ligands of folic acid for the coating. For evaluating anticancer and apoptosis effects, we assessed changes in important genes and proteins in apoptosis (p53, caspase-3, Bax, Bcl-2) in several cell lines (MCF-7, breast adenocarcinoma; HCT-116, colon carcinoma; HOS, human osteosarcoma; and A-549, non–small cell lung cancer). Results: Nanoformulations were successfully synthesized and contained 10.9 wt.% for the CL single-delivery version and 18.1 wt.% for the CL+CR co-delivery nanoformulation. Anticancer effects depended on treatment, cell line, and concentration. Co-delivery nanoformulations exerted anticancer effects that were significantly superior to those of single delivery or free CL or CR. Anticancer effects by cell line were in the order of HCT-116 > A549 > HOS > MCF-7. The lowest IC50 value was obtained for the nanoformulation consisting of CL and CR coated with a polymeric shell conjugated with FA (equivalent to 4.1 ± 0.05 µg/mL). With dual delivery compared with the free agents, we detected strongly increased p53, caspase-3, and Bax expression, but inhibition of Bcl-2, suggesting promotion of apoptosis. Conclusions: Our findings, although preliminary, indicate that the proposed dual delivery nanoformulation consisting of nanocore: MSNs loaded with CL and CR and coated with a shell of chitosan–cellulose conjugated folic acid exerted strong anticancer and apoptotic effects with potent antitumor activity against HCT-116 colon cells. The effect bested CL alone. Evaluating and confirming the efficacy of co-delivery nanoformulations will require in vivo studies.

## 1. Introduction

A co-delivery nanosystem (CDNS) consists of nanoformulations based on nanoparticles (i.e., a nanomedicine platform) and two or more pharmaceutical agents for their targeted delivery to selected cells. This construct has received considerable attention recently because it offers potential advantages in the treatment of disease, including cancers [1,2]. Such systems have shown superior therapeutic efficacy against cancers and have been accepted as an applicable strategy in clinical practice in recent decades [3,4]. Among their many advantages in cancer therapy are overcoming multidrug resistance [5] and modulating different signaling pathways/mechanisms, which is likely to promote a synergistic effect [1]. As a consequence, combination therapy can enhance drug effects and reduce the required drug dose, leading to decreased side effects and toxicity [6,7]. In addition, the loading of multiple pharmaceutical agents with different characteristics may produce better anticancer effects than delivery of a single pharmaceutical agent [3]. The feasibility of using the CDNS approach has been assessed by comparisons with single-loading and free-drug forms. For instance, methoxy PEG-PLGA nanoparticles with a core shell that are loaded with doxorubicin and paclitaxel together exert stronger anticancer effects because of a synergism between them when compared with delivery of either, singly [8]. A CDNS consisting of etoposide and curcumin and using lipid nanoparticles allowed for synergism of the drugs, resulting in impressive anti-tumor effects, which could improve clinical outcomes in cancer therapy [9].

The nanocarrier is a key factor in determining CDNS success. Mesoporous silica nanoparticles (MSNs) are inorganic-based nanocarriers and among the most investigated nanomedicine platforms as systems for drug delivery (DDSs). Features of MSNs include a high surface area, large size, and large pores, suitable for surface modifications, high loading capacity, multifunctional cancer targeting, and possible large-scale synthesis. Thus, they are an ideal nanomedicine platform and have been used in DDSs targeting cancers. Among other groups, we have published findings using several DDSs and various kinds of MSNs against cancer cells [10,11,12]. Recently, the construction of DDSs with MSNs for a diverse range of anticancer chemotherapeutic combinations (such as phototherapy, gene delivery, and immunotherapy) has led to anticipated features, including a synergistic effect and significant improvement in cancer-related therapeutic outcomes [13]. Combination therapy with various designs based on MSNs has been described [14,15,16,17], showing efficient anticancer improvements. For instance, folic acid (FA), a small molecule, influences essential biological effects such as production of nucleic acid, division of cells, and metabolic processes in cells [18,19]. It is known that folate receptor is overexpressed in the most cancer types with increased density as cancer becomes worsens [19]. Therefore, FA is effectively used as a ligand molecule to design various delivery systems with MSNs for cancer targeting [10,20,21].

Curcumin (CR) is one of the most effective natural agents employed for combination therapy. It is a polyphenolic compound that takes its name and is derived from the *Curcuma longa* plant. Evidence hints that CR shows several pharmacological activities associated with good safety. Additionally, it has been identified as acting via multifunctional anticancer mechanisms, and thus might be likely to improve therapeutic effects when combined with a variety of drugs [22] or loaded to MSNs [23]. For example, a combination of CR with the anticancer drug sunitinib exerted a strong synergistic effect against MCF-7 breast cancer cells and increased effectiveness relative to sunitinib alone in an animal model [24]. Findings from animal studies support the superior efficiency of CR combination-based nanoformulations against cancers, with studies showing benefit of CR plus doxorubicin for liver cancer [25] and CR plus 5-FU for liver cancer [26]. Furthermore, the success of CR combined with chemotherapy for cancer has been confirmed across many clinical trials: with docetaxel for breast cancer (advanced, metastatic) [27], gemcitabine for pancreatic malignancies [28], imatinib for chronic myeloid leukemia [29], and oxaliplatin for colorectal liver metastases [30]. Although nanoformulation-based combination anticancer therapy with CR has received much attention, especially in combination with chemotherapy, nanoformulations that combine CR with natural agents for cancer therapy are lacking.

**Scheme 1:**
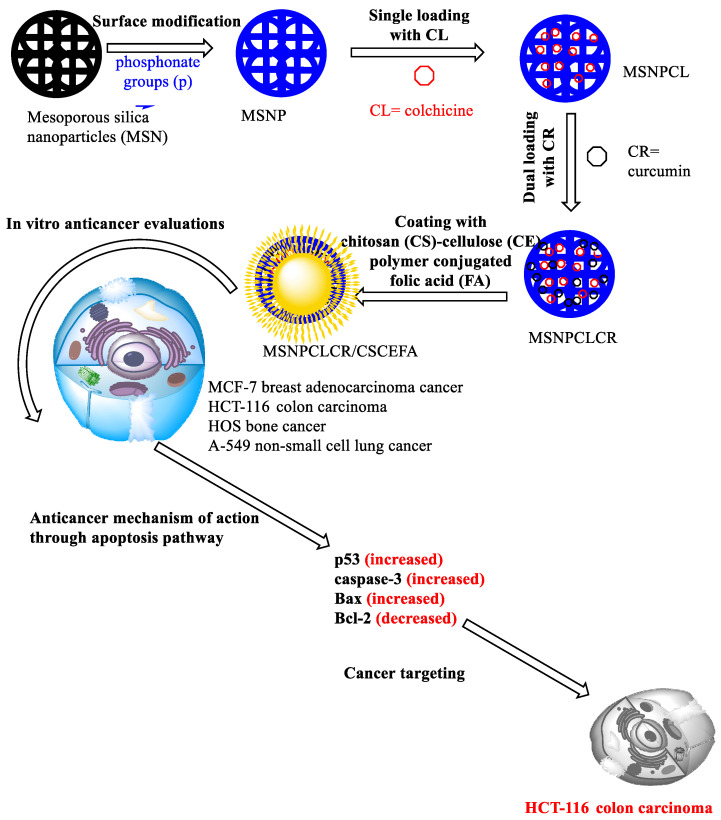
Preparation of the dual delivery system from single to dual-based nanoformulations with CL and CR natural agents showing the anticancer evaluations on MCF-7, HCT-116, HOS, and A549 cancer cell lines and the apoptosis biomarkers p53, caspase-3, Bax, and Bcl-2.

In this study, we fabricated a CDNS for colchicine (CL) and CR made of phosphonate-functionalized MSNs carrying both agents as a core and bearing a polymeric coating of chitosan (CS)-cellulose (CE) conjugated with a folic acid (FA)-targeting ligand (as coating shell), as shown in Scheme 1. To the best of our knowledge, the combination of curcumin with colchicine as anticancer model natural compounds loaded in mesospheres has not been studied yet. Our findings indicate enhanced anticancer effects with the combination, which proved to be efficiently selective for killing colon cancer cells. To our knowledge, the co-delivery of CR and CL in a system using drug-loaded MSNs for cancer treatment has not been reported previously. The current findings suggest that this construct could improve anticancer therapeutic efficiency of CL while decreasing its toxicity to normal cells, which to date has prevented its clinical use [31,32,33,34,35].

## 2. Materials and Methods

### 2.1. Synthesis of Phosphonate-Functionalized MSNs

MSNs were synthesized as previously described [12]. The obtained MSN powder was used to synthesize phosphonate functionalized to improve the CL loading content MSNs according to our previous report [11]. Briefly, 1.5 g MSN was suspended in deionized water under stirring, followed by the addition of 2 mL of a salt solution (3-(trihydroxysilyl)propyl methylphosphonate monosodium; Santa Cruz Biotechnology, Dallas, TX, USA), and maintained at room temperature for 24 h. After centrifugation, the solution was washed repeatedly in deionized water, followed by oven-drying for 24 h at 60 °C. The resulting powder was designated as MSNP nanocarrier.

### 2.2. Fabrication of Nanoformulations

#### 2.2.1. Core Nanoformulations

For CL single loading to MSNP, we experimentally used a 1:3 loading ratio (CL:MSNP) as described in the following steps: dissolving of 200 mg CL (Sigma-Aldrich, St. Louis, MO, USA) in deionized water (10 mL) under stirring, followed by addition of 600 mg MSNP and stirring 24 h at room temperature. For collection of the loaded nanoparticles, the mixture solution was centrifuged and washed with deionized water (2×) to remove unloaded CL molecules. The collected loaded nanoparticles were oven-dried at 60 °C. The obtained powder was designated as the MSNPCL core nanoformulation.

For the CL and CR combination, we began with the prepared MSNPCL nanoformulation, resuspending 300 mg in ethanol containing 100 mg CR, and then stirred for 24 h at room temperature. The resulting product was centrifuged for collection and designated as the MSNPCLCR core nanoformulation.

#### 2.2.2. Core-Shell Nanoformulation Preparation

To obtain the CS-CE polymer mixture solution, we dissolved 250 mg CS (Acros Organics, Geel, Belgium) in 20 mL acetic acid (2%), followed by 2 h of stirring at 60 °C (solution A). A solution of 10 mL acetone containing 100 mg CE, made with the assistance of sonication (solution B), was slowly added to solution A and then kept under stirring for 10 h to yield a CS-CE polymer mixture solution. We prepared the activated FA solution by adding 85 mg FA, 70 mg 1-(3-dimethylaminopropyl)-3-ethylcarbodiimide hydrochloride (Acros Organics), 50 mg N-hydroxysuccinimide (Acros Organics), and 0.250 mL triethanolamine (Molekula GmbH, Munich, Germany) dissolved in 20 mL dimethyl sulfoxide (Tedia, Fairfield, OH, USA) under stirring for 20 h at room temperature. To obtain CS-CE–conjugated FA, the FA activated solution was dropped into the CS-CE polymer mixture solution under stirring for 4 h at 50 °C, resulting in a CS-CE polymer–conjugated FA solution. This solution was maintained at −20 °C. To yield the core-shell nanoformulation, we resuspended MSNPCLCR in 20 mL CS-CE polymer–conjugated FA solution under stirring (medium stirring 250 rpm) for 24 h, followed by centrifugation, washing with deionized water, and complete drying at 60 °C in an oven. The resulting product was designated as the MSNPCLCR/CSCEFA core-shell nanoformulation. All materials were maintained at room temperature.

### 2.3. Material Characterization

#### 2.3.1. Instrumentation and Measurements

##### Electron Microscopy

For viewing the structure of the prepared nanoparticles and nanoformulations, we used transmission electron microscopy (TEM) or scanning TEM (STEM) with a FEI TECNAI G2 F20 S-TWIN (Thermo Fisher Scientific, Waltham, MA, USA).

##### X-ray Diffraction (XRD)

To determine the crystalline structure of materials at all stages of preparation, we applied powder XRD (X’PertPRO System, PANalytical, Marietta, GA, USA). The conditions for this characterization were CuKα radiation with a 10°–100° 2θ range.

##### Surface Area

For measuring the surface area of nanoparticles and nanoformulations, we used specific surface area analysis (Brunauer, Emmett, and Teller; Gemini 2360, Micromeritics, Norcross, GA, USA; ISO 9277:2010). The conditions were as follows: MSNP powders were dried (150 °C), and the powder of nanoformulations composed of natural agents were dried 24 h at 50 °C under constant flow of helium (FlowPrep 060 desorption station, Micromeritics).

##### Fourier Transform Infrared (FTIR) Spectroscopy

For characterization of functional groups on the nanoparticle and nanoformulation surfaces, we applied FTIR spectroscopy analysis (Bruker Optics Tensor 27, Bruker Corporation, Billerica, MA, USA) through attenuated total reflectance (ATR) (Platinum ATR-Einheit A 255).

##### Thermal Properties

We characterized the thermal properties of the materials using simultaneous thermal analysis (STA)–coupled differential scanning calorimetry (DSC) analysis (STA 499 F1 Jupiter; NETZSCH-Feinmahltechnik GmbH, Selb, Germany) in the following steps: a pre-measurement sample weight of ~10–17 mg was inserted into the alumina pan, with helium flow through the STA furnace chamber for 30 min. The measurement condition was programmed by reaching 800 °C, at a rate of 10 °C per min under a helium/air mixture.

##### Zeta Potential

To determine zeta potential, we used a Malvern ZetaSizer (NanoZS, Malvern, UK) to obtain the surface charges of the materials in water suspension at room temperature at all stages.

### 2.4. In Vitro Cytotoxicity Evaluation

For cytotoxicity, viability was assessed in 96-well culture plates using the MTT assay (i.e., (3-(4, 5-dimethylthiazol-2-yl)-2,5-diphenyl tetrazolium bromide). We used four cell lines: MCF-7 breast adenocarcinoma, HCT-116 colon carcinoma, HOS human osteosarcoma, and A-549 non–small cell lung cancer (Karolinska Institute, Stockholm, Sweden), maintained in RPMI 1640 supplemented with 10% heat-inactivated fetal bovine serum plus 1% antibiotic–antimycotic mixture (10,000 µg/mL streptomycin sulfate, 25 µg/mL amphotericin B, 10,000 U/mL potassium penicillin, and 1% L glutamine; Biowest, Riverside, MO, USA). BJ-1 human skin fibroblasts derived from foreskin (American Type Culture Collection, CRL-2522) were maintained in DMEM (consisting of 2 mM L-glutamine, Earle’s salts medium) (Biowest). Our experiments were performed in a sterile laminar air flow cabinet of biosafety class II. All incubations took place at 37 °C under 5% CO_2_ in a 95% humidified atmosphere.

Cells were seeded in 96-well microtiter plastic plates to a density of 10^4^ cells/well and incubated for 24 h. After the medium was aspirated, we added fresh medium containing MSNP (up to 100 µg/mL), nanoformulations (equivalent concentration of CL or CR nanoformulations up to 100 µg/mL), or free CL and CR (up to 100 µg/mL). The equivalent concentration in nanoformulations was prepared based on natural prodrug content in nanoformulations calculated from the weight loss values: 10.9 wt.% for MSNPCL and 18.1 wt.% for MSNPCLCR and MSNPCLCR/CSCEFA. Cells were incubated with treatments for 48 h, with DMEM alone for untreated control cells. Afterward, 40 µL MTT salt (Bio Basic Canada Inc., Toronto, ON, Canada) at 2.5 µg/mL per well was added, followed by a 4 h incubation. The addition of 200 µL of 10% sodium dodecyl sulfate (SDS) and overnight incubation at 37 °C was used to stop the reaction and dissolve formazan crystals. Formazan product was measured on a microplate reader at 595 nm to 690 nm (reference wavelength) as background (model 3350, Bio-Rad, Hercules, CA, USA). The calculation for cytotoxicity was [(reading of extract/reading of negative control) − 1] × 100. The IC50 (concentration yielding 50% inhibition of cell viability) was calculated by applying various concentrations of treatments and the probit analysis method with a *t*-test (SPSS version 11.0, Chicago, IL, USA).

### 2.5. RT-qPCR Gene Expression Analysis

We profiled the expression of pro- and anti-apoptosis markers in HCT-116, HOS, A549, and MCF-7 cells exposed for 72 h to IC50 doses of the treatments. In brief, treated cells were collected and total RNA isolated per instructions (total RNA Purification Kit; Norgen Biotek Corp., Thorold, ON, Canada). One microgram purified RNA was used to synthesize cDNA in a final reaction volume of 20 µL using the QuantiTect Reverse Transcriptase Kit (Qiagen, Hilden, Germany). Thermal programs were conducted on a Rotor-Gene Q 5-Plex HRM thermal cycler (Qiagen, Germany) and PCR amplicon specificity was confirmed using QuantiTect SYBR-Green PCR Kit (Qiagen, Germany) as previously described and following standard protocols [36]. Forward and reverse primers were as follows: Bax, 5′-AAGCTGAGCGAGTGTCTCCGGCG-3′ and 5′-CAGATGCCGGTTCAGGTACTCAGTC-3′; B-cell lymphoma 2 (Bcl-2), 5′-CTCGTCGCTACCGTCGTGACTTGG-3′ and 5′-CAGATGCCGGTTCAGGTACTCAGTC-3′; p53, 5′-GCTCTGACTGTACCACCATCC-3′ and 5′-CTCTCGGAACATCTCGAAGCG-3′; caspase-3, 5′-CAAACTTTTTCAGAGGGGATCG-3′ and 5′-GCATACTGTTTCAGCATGGCA-3′; and glyceraldehyde 3-phosphate dehydrogenase (GAPDH) internal control, 5′-CGGAGTCAACGGATTTGGTC-3′ and 5′-AGCCTTCTCCATGGTCGTGA-3′. The reaction mixture had a final volume of 25 µL (5 µL diluted cDNA sample, 12.5 µL 2× SYRB-Green PCR Master Mix, 10 µM stock/2.5 µL of each primer, and 2.5 µL RNase-free water). For each sample, we included by three biological and three technical replicates, and a no-template control. To analyze raw data, we used Rotor-Gene^®^ 2.1 (Qiagen GmbH, Düsseldorf, Germany) to calculate the threshold cycle (Ct) using the second derivative maximum. After normalization to GAPDH expression, the fold-change value for each gene was calculated using 2^−ΔΔCt^.

### 2.6. Western Blot Analysis

Immunoblotting analysis was carried out as previously described [37]. After 72 h of IC50 treatment, cells were collected and homogenized in protein extraction buffer (Roche). Extracts were centrifuged twice at 13,000× *g* for 10 min at 4 °C. Supernatants were transferred to new tubes and the protein content quantified (Bradford assay; Bio-Rad). For each sample, 20 µg of each protein extract was mixed with 2× loading buffer, heated at 95 °C, and separated on 12% SDS-PAGE gels. After transfer of gels, a polyvinylidene fluoride membrane, the membrane was blocked with 5% non-fat milk diluted in phosphate-buffered saline containing 0.1% Tween-20. Target proteins were immunodetected with the respective primary antibody anti-Bax E63 monoclonal, ab32503; (anti-Bcl-2 monoclonal, E17, ab32124; anti-Caspase-3 polyclonal, ab13847; anti-β actin monoclonal, SP124, ab115777; anti-p53 monoclonal, PAb 240, ab26; all Abcam, Cambridge, UK). As secondary antibodies, we used anti-mouse IgG (Amersham Biosciences, Buckinghamshire, UK) and anti-rabbit IgG (GE Healthcare, Milwaukee, WI, USA).

### 2.7. Statistical Analysis

Results of analyses of biological data are given as mean ± standard deviation (SD). Significant differences for anticancer effects were evaluated by one-way analysis of variance (ANOVA; GraphPad PRISM, v 8.0.1, San Diego, CA, USA). Analysis of RT-PCR results was conducted by one-way ANOVA in rapid publication-ready MS Word tables according to [38].

## 3. Results

### 3.1. Morphological Structure

**Figure 1:**
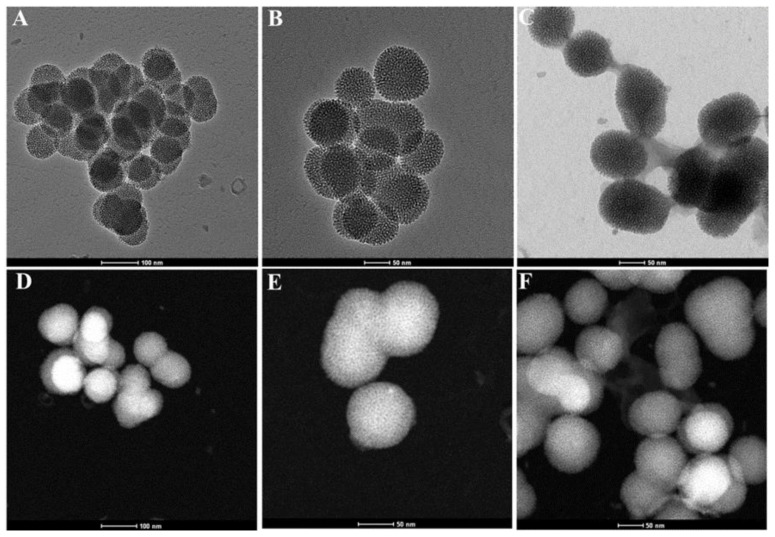
STEM images for nanoformulations before and after polymeric-FA coating showing the morphological changes. (**A**) TEM image: MSN. (**B**). TEM image: MSNPCLCR. (**C**) TEM image: MSNPCLCR/CSCEFA. (**D**) STEM image: MSN. (**E**) MSNPCLCR. (**F**) MSNPCLCR/CSCEFA.

Figure 1 shows the morphological structural changes after coating, by TEM and STEM. As shown in the TEM images, MSN and MSNPCLCR particles were uniform spherical shapes with a tendency to aggregate (Figure 1A,B). Following the attachment of the CS-CE-FA coating in MSNPCLCR/CSCEFA, a transparent layer was formed on the surface of these particles (Figure 1C). Similarly, STEM images confirmed the coating formation in the comparison of MSN and MSNPCLCR (Figure 1D,E) with MSNPCLCR/CSCEFA (Figure 1F). The coating layer was not seen in some places under the observation field.

### 3.2. XRD Characterization

**Figure 2:**
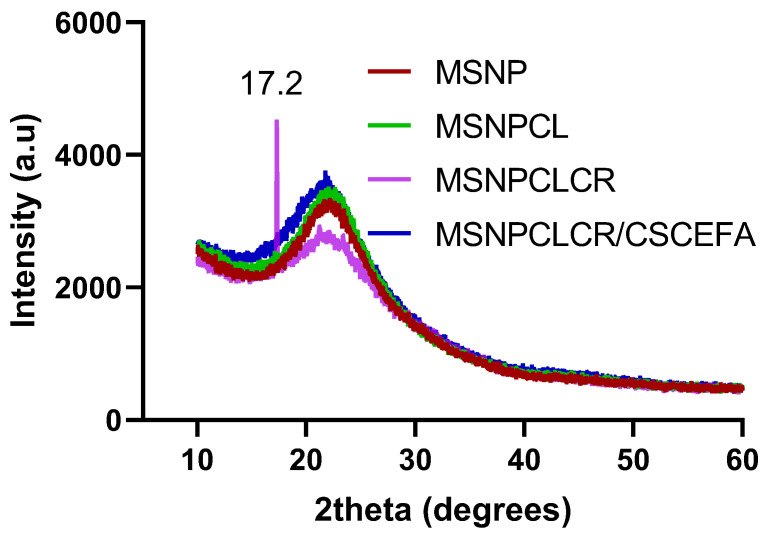
The XRD patterns for MSNP and nanoformulations before and after the coating with CS-CE-FA conjugation. One crystalline peak at 2θ: 17.2 is related to CR loading; the peak disappeared after coating.

The XRD patterns for MSNP and nanoformulations are shown in Figure 2. Few changes were observed related to shifting in the patterns for MSNP, MSNPCL, MSNPCLCR, and MSNPCLCR/CSCEFA, centered at ~22° and characterizing the amorphous phase of the silica framework. Only one crystalline peak at 17.2° was observed for MSNPCLCR, corresponding to the attachment of a small fraction of CR molecules [10] presenting on the surface, as compared with the MSNPCL (CL alone). Of note, this peak disappeared after polymer coating, as seen in MSNPCLCR/CSCEFA. This observation particularly confirmed the successful shell coating/embedding, in agreement with observations from the TEM and STEM images (Figure 1).

### 3.3. STA-DSC Characterization

**Figure 3:**
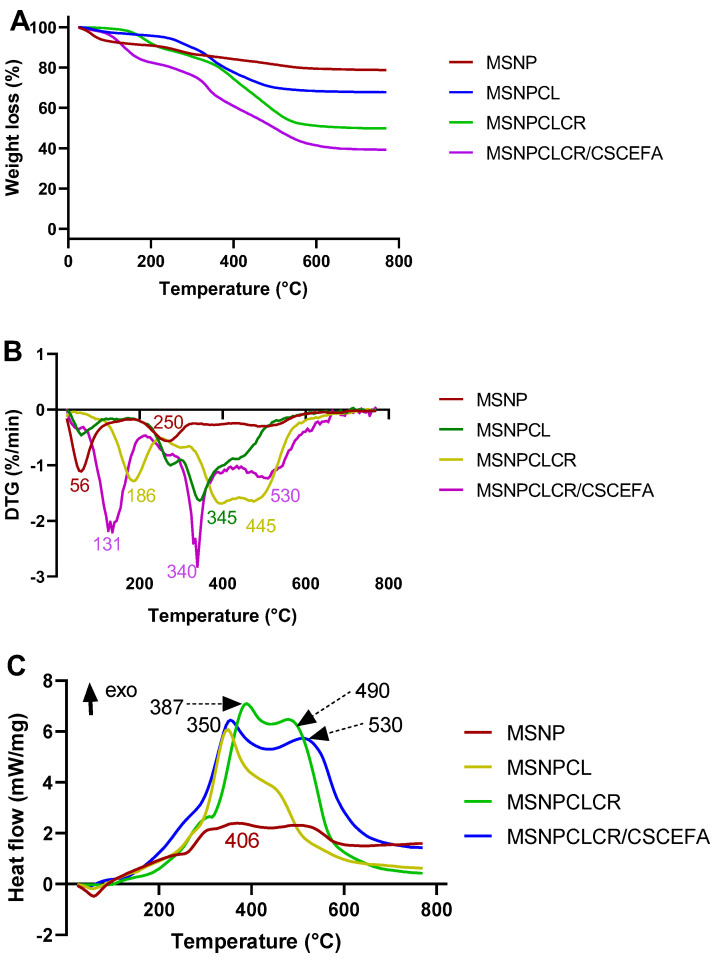
Thermal properties of MSNPs, nanoformulations, and free natural agents by STA–DSC analysis. (**A**) The mass loss analysis by STA at all stages. (**B**) DTG patterns at all stages according to mass loss. (**C**) The differential scanning calorimetry. (DSC) Thermograms at all stages.

After all of the materials were heated to ~770 °C, we observed a great change in mass loss (wt.%). The weight loss varied from 21.5 wt.% (MSNP) to 32.4 wt.% (MSNPCL), 50.5 wt.% (MSNPCLCR), and 60.9 wt.% (MSNPCLCR/CSCEFA), respectively, ascribed to the surface modification with phosphonate groups, CL loading, co-loading of CR and CL, and coating with polymer mixtures (Figure 3A, Table 1). The weight loss of MSN (data are not shown) about 15.5 wt.%, so phosphonate content in MSNP was about 6 wt.%. The calculated total prodrug content (TPC, wt.%) was 10.9 wt.% for MSNPCL, 18.1 wt.% for MSNPCLCR, and 18.1 wt.% for MSNPCLCR/CSCEFA. The polymeric shell content was 10.3 wt.%. There was a broad peak at 280 °C for MSNP, resulting from phosphonate group decomposition (Figure 3B). The DSC curves presented a broad peak centered at 406 °C for MSNP (Figure 3C). In nanoformulations, DTG spectra displayed several broad peaks centered at 345 °C (MSNPCL), 186 °C and 445 °C (MSNPCLCR), and 131 °C, 340 °C, and 530 °C (MSNPCLCR/CSCEFA) (Figure 3B). All of these peaks were attributed to the decomposition of organic content—both CL and CR and polymeric substances in the case of coating, which takes place through stages corresponding to heading temperature. The first stage occurred from room temperature to 200 °C, and the second stage occurred from 300 °C to 600 °C. In agreement with the DTG results, the main changes in DSC curves (Figure 3C) were detected in the 300 °C to 600 °C range for nanoformulations. MSNPCL resulted in an exothermic peak centered at 350 °C, MSNPCLCR had exothermic peaks at 387 °C and 490 °C, and MSNPCLCR/CSCEFA had exothermic peaks at about 350 °C and 530 °C. These peaks in nanoformulations likely are the result of their shifting from the location of the original peaks associated with the free natural agents. The shifted peaks could indicate the attachment of some number of free agents to the surface in a crystalline form, in agreement with the XRD results obtained for MSNPCLCR, which presented a sharp crystalline peak at 17.2°.

### 3.4. FTIR-ATR Characterization

**Figure 4:**
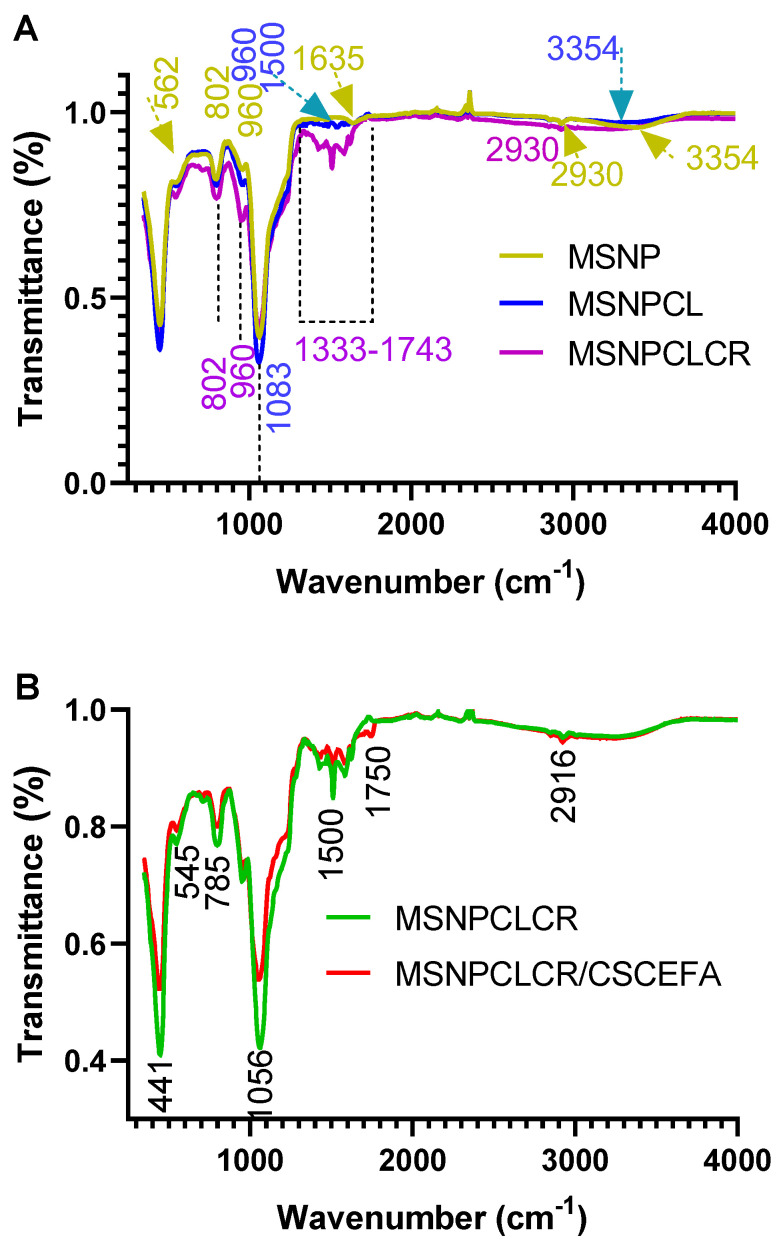
FTIR–ATR spectra of the materials prepared at all stages: MSNP, nanoformulations before and after CS–CE–FA complex coating. (**A**) nanoformulations before coating, and (**B**) MSNPCLCR before coating compared with MSNPCLCR/CSCEFA after coating.

FTIR-ATR spectra showed obvious changes in surface-modified nanoparticles and nanoformulations (Figure 4A). In MSNP, several peaks were shifted, intensified, or new, as seen at 562, 802, 960, 1635, 2930, and 3354 cm^−1^, which strongly confirmed the surface modification compared with MSNs [39]. Concerning nanoformulations, the spectra obtained for MSNPCL showed a clearly shifted peak at 960 cm^−1^, a new peak at 1600 cm^−1^, and a peak at 3354 cm^−1^ that was slightly less intense compared with MSNP. Further CR loading, as indicated by MSNPCLCR, resulted in more intensive peaks at 802 and 960 cm^−1^, new peaks in the region between 1333 to 1743 cm^−1^, and broader peaks centered at 2930 cm^−1^. These peaks could have corresponded to the presence of loaded CR. For the changes before and after polymeric coating, Figure 4B shows the changes between MSNPCLCR and MSNPCLCR/CSCEFA in several positions. The intensive peaks at 441, 545, 785, 1056, 1500, 1750, and 2916 cm^−1^ in MSNPCLCR were slightly lower in MSNPCLCR/CSCEFA because of the polymeric coating.

### 3.5. Zeta Potential Measurement

**Figure 5:**
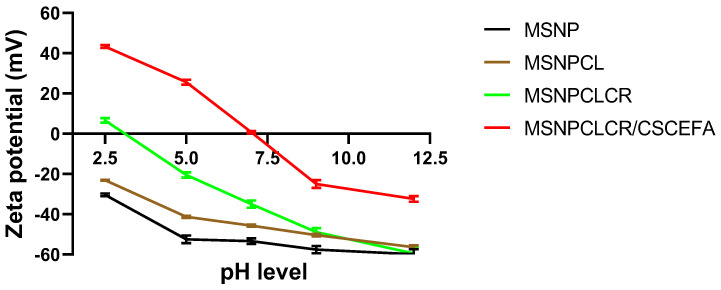
Zeta potential measurement of MSNP and nanoformulations before and after coating, suspended in deionized water. The zeta potential was recorded for the prepared materials at different pH levels presenting the range of low acidic to basic media.

Figure 5 depicts the changes in zeta potential values for MSNP and nanoformulations suspended in deionized water related to changes in pH. Both MSNP and MSNPCL were negatively charged in acidic, neutral, and alkaline media, and increasing pH to 12.5 resulted in high negative zeta potential of −59.9 ± 2.5 mV and −56.5 ± 0.9 mV, respectively. MSNPCLCR showed only a positive zeta potential of 6.6 ± 1.0 mV at pH 2.5, but the zeta potential turned negative and reached ~−60 ± 2.8 mV at pH 12.5. MSNPCLCR/CSCEFA showed a different pattern with a positive zeta potential of 43.3 ± 0.7, 25.6 ± 1.2, and 0.7 ± 0.6 at pH 2.5, 5, and 7 (from acidic to neutral), respectively. As the medium became alkaline, there was a shift, to −24.9 ± 1.8 and −32.4 ± 1.4 at pH 9 and 12, respectively.

### 3.6. In Vitro Anticancer Effects

**Figure 6:**
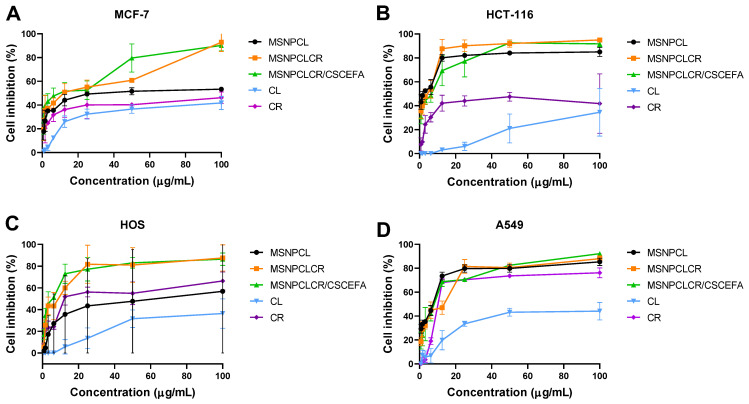
The anticancer evaluation of nanoformulations compared with free CL and CR against cancer cells treated at different concentrations (µg/mL) incubated for 48 h. (**A**) MCF7 breast cancer cells, (**B**) HCT-116 colon cancer cells, (**C**) HOS bone cancer, and (**D**) A549 lung cancer cells. Data are presented as mean ± SD. The concentration was prepared for nanoformulations as an equivalent concentration of CL and CR in nanoformulation equal to free CL and CR.

As Figure 6 shows, the anticancer effect increased with increasing treatment concentration in all four cancer cell lines. Nanoformulations compared with free CL and CR tended to be the most significantly effective treatments (*p* < 0.05), inhibiting all four cell line cells. MSNPCLCR and MSNPCLCR/CCFA generally had better anticancer effects than MSNPCL, which was expected. Free CR had a greater anticancer effect than free CL on all cells. Figure 6A shows that MSNPCLCR/CCFA, MSNPCLCR, and MSNPCL produced the highest anticancer effect at >90% (no significant differences among these nanoformulations), followed by CR (~47%) and CL (~42%). As shown in Figure 6B, the most effective treatments against HCT-116 were nanoformulations compared with free CL or CR. Despite a lack of significant differences among the nanoformulations, their anticancer potential was ordered as follows: MSNPCLCR > MSNPCLCR/CCFA > MSNPCL. Similarly, as shown in Figure 6C, MSNPCLCR and MSNPCLCR/CCFA exhibited the highest anticancer activity against HOS cells compared with MSNPCL, CR, and CL. We found no significant difference between CR and MSNPCR. As Figure 6D shows, there were no significant differences among the treatments, but in A549 cells, free CL showed the lowest anticancer effect compared with other treatments.

Table 2 shows that nanoformulations efficiently inhibited cancer cells compared with free CL and CR, as indicated by their lowest IC50. Both MSNPCLCR and MSNPCLCR/CSCEFA tended to be more efficient than MSNPCL at killing cancer cells. Of the cell lines, HCT-116 was most sensitive to treatment, followed by A549, HOS, and MCF-7. In a comparison of free CL and free CR, CR had the higher anticancer effect, as indicated by its lower IC50.

Taken together, the results indicate that, compared with free CL and CR, the nanoformulations, especially MSNPCLCR and MSNPCLCR/CCFA, showed better anticancer activity and superior killing of HCT-116 over the MCF-7, HOS, and A549 cell lines. The data also demonstrate that dual-delivery nanoformulations were efficient compared with single-delivery nanoformulation. This observation is closely connected with the lowest anticancer potential of free CL versus free CR, as indicated in all four cancer line results.

### 3.7. Apoptosis Induction Evaluations

#### 3.7.1. Protein Expression by RT-PCR

**Figure 7:**
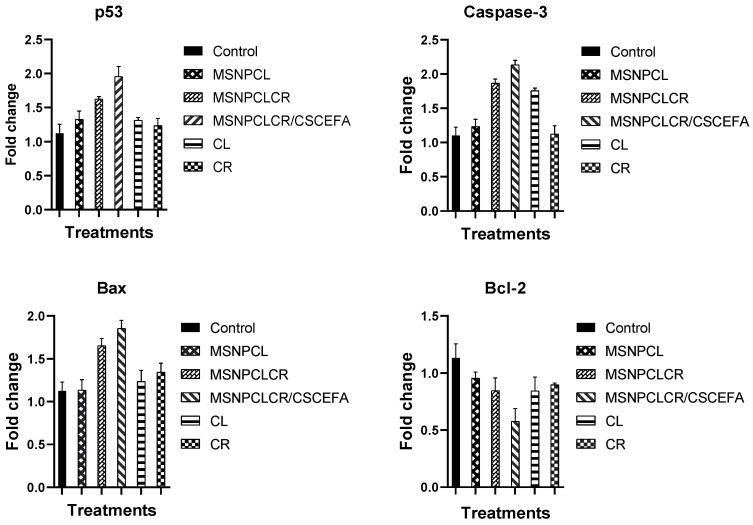
RT–PCR values for p53, caspase-3, Bax, and Bcl-2 markers of apoptosis for nanoformulations compared with free CL and CR against MCF-7 cancer cells treated at the IC50 concentration (µg/mL) of each formulation by incubation for 72 h. Data are presented as mean ± SD. The concentration was prepared for nanoformulations as an equivalent concentration of CL and CR in the nanoformulation equal to free CL and CR.

**Figure 8:**
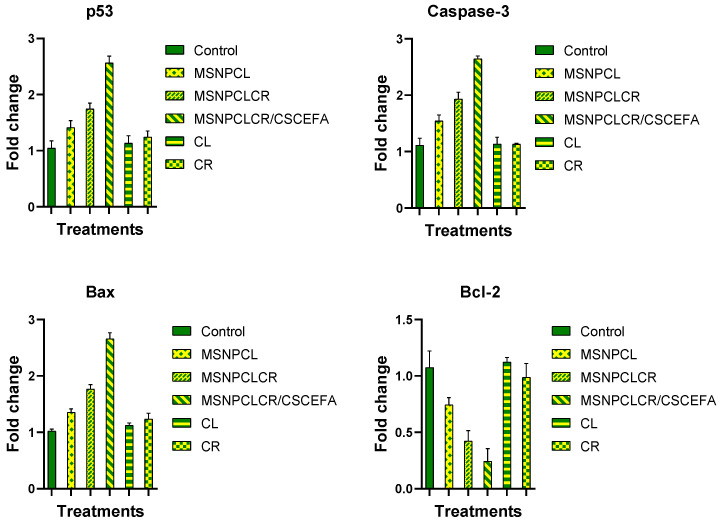
RT–PCR measurements of p53, caspase-3, Bax, and Bcl-2 markers of apoptosis for nanoformulations compared with free CL and CR against HCT-116 cancer cells treated at the IC50 concentration (µg/mL) of each formulation by incubation for 72 h. Data are presented as mean ± SD. The concentration was prepared for nanoformulations as an equivalent concentration of CL and CR in the nanoformulation equal to free CL and CR.

**Figure 9:**
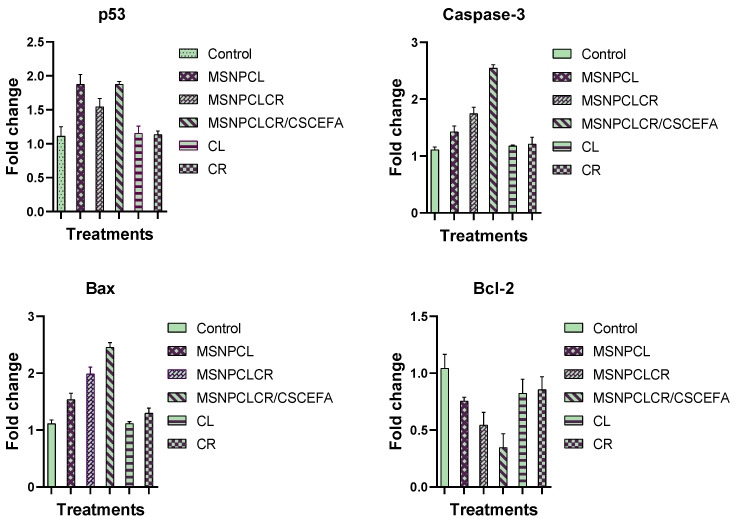
RT–PCR measurements of p53, caspase-3, Bax, and Bcl-2 markers of apoptosis for nanoformulations compared with free CL and CR against HOS cancer cells treated at the IC50 concentration (µg/mL) of each formulation by incubation for 72 h. Data are presented as mean ± SD. The concentration was prepared for nanoformulations as an equivalent concentration of CL and CR in the nanoformulation equal to free CL and CR.

To identify a potential mode of action for the growth inhibitory effects of nanoformulations and free natural agents against cancer cell lines (HCT-116, MCF-7, HOS), we examined expression of key apoptosis genes. We observed potential enhanced effects of nanoformulations compared with free CL and CR on the three cancerous cell lines after a 72 h exposure to IC50 concentrations (MCF-7, Figure 7; HCT-116, Figure 8; HOS, Figure 9). The most effective treatments were detected in this order: MSNPCLCR/CSCEFA > MSNPCLCR > MSNPCL > CR > CL. The pro-apoptosis p53, Bax, and caspase-3 were significantly upregulated, and the anti-apoptosis Bcl-2 was downregulated in all cancer cells. Of note, a high apoptosis induction rate was observed in HCT-116 cells compared with MCF-7 and HOS. As indicated from the results, MSNPCLCR/CSCEFA was the most effective treatment for HCT-116, which showed elevated expression of p53 (~2.5 ± 0.12 fold change), caspase-3 (~2.6 ± 0.05 fold change), and Bax (~2.6 ± 0.11 fold change) and decreased expression of Bcl-2 (by ~0.24 ± 0.11 fold).

#### 3.7.2. Protein Expression by Western Blotting

**Figure 10:**
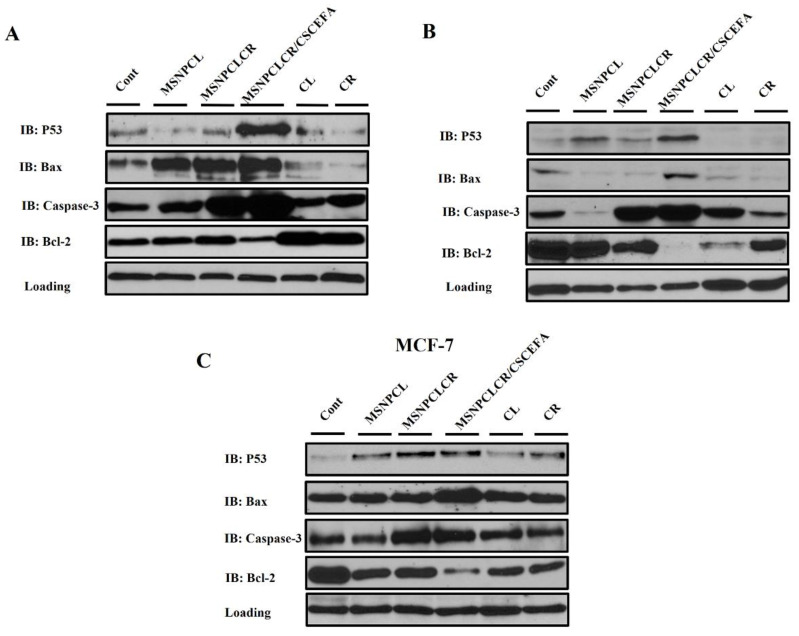
Western blot analysis of p53, caspase-3, Bax, and Bcl-2 markers of apoptosis for nanoformulations compared with free CL and CR against HOS cancer cells treated at the IC50 concentration (µg/mL) of each formulation by incubation for 72 h. The concentration was prepared for nanoformulations as an equivalent concentration of CL and CR in nanoformulation equal to free CL and CR. (**A**) western blot analysis for HCT-116 cancer cells; (**B**) western blot analysis for HOS cancer cells; (**C**) western blot analysis for MCF-7 cancer cells.

To further explore potential mechanisms, we used immunoblotting with specific antibodies against p53, caspase-3, Bax, and Bcl-2 to trace the path of the cell death program in MCF-7, HCT-116, and HOS cell lines after 72 h of incubation with treatments. The results indicated that p53, caspase-3, and Bax proteins increased in the three cell lines exposed to treatment with the IC50 of MSNPCR/CSCEFA compared with MSNPCL, MSNPCLCR, CL, and CR (Figure 10). Levels of the pro-apoptosis Bcl-2 protein were reduced or undetected with MSNPCR/CSCEFA treatment relative to untreated controls. These results indicated that, compared with controls, the MSNPCR/CSCEFA nanoformulation had more potential for apoptosis induction against HCT-116, followed by HOS and MCF-7 cells.

## 4. Discussion

Improvement in the anticancer activity of natural agents is important not only to enhance safety but also to move them towards clinical application. The integration of drug delivery and nanotechnology for cancer therapy has gained considerable attention [40,41,42,43]. Among these delivery systems, interest has been growing in the combination of cancer therapy with targeted delivery system-based nanoformulations [44,45,46]. In the current study, we used functionalized MSNs with phosphonate groups as ideal inorganic nano-vehicles characterized by high drug-loading capacity. We attempted to plan a core-shell nanoformulation employing functionalized MSNs for targeted delivery of both agents, first loading CL and then using the loaded nanoparticles to further load CR. We hypothesized that with this approach, CR would release first, followed by CL, which is less toxic. This method offers an alternative to the use of a mixture of anticancer drugs loaded directly with MSNs, such as paclitaxel and curcumin with the PEGylated lipid bilayer–coated MSNs [47]. After we co-loaded natural agents into functionalized MSNs, we developed a coating shell of a CS-CE polymer mixture conjugated with cancer-targeting ligands of FA. The core-shell co-delivery system with MSN has been achieved with another design for doxorubicin/paclitaxel co-delivery [45]. In that system, paclitaxel was covalently attached to MSN-loaded doxorubicin, and then polystyrene sulfonate was electrostatically coated through a microfluidic technique. The use of the CS-CE coating in our system can gate the release rate of natural anticancer agents, providing a sustained release pattern and permitting active cancer targeting via the interaction of FA with folate receptors available on the cancer cell membrane. Of note, one of the most reported mechanisms by which both natural agents kill cancer cells is through apoptosis activation (programmed cell death) [48]. Among the many factors involved in the apoptosis pathway, we chose p53, caspase-3, Bax, and Bcl-2 to test in cancer cells. These molecular and cellular pathways for targeting apoptosis have been reported for CR and CL [32,49,50,51].

The results of several analytical techniques used to characterize the physicochemical properties of nanoformulations confirm the successful formulations of the proposed dual delivery system. The morphological changes seen in TEM and STEM images confirmed the CS-CE polymer mixture coating shell in MSNPCLCR/CSCEFA. This observation agrees with our previous findings using core-shell nanoformulations with a mixture of different polymers to deliver a thymoquinone prodrug with MSNs [12]. Similarly, in our previous study, the nanoformulation comprised CL-loaded MSNs functionalized with phosphonates coated with chitosan-glycine conjugated to FA [11]. XRD and DSC characterization of nanoformulations revealed that a small fraction of loaded CR with CL may be presented on the surface at the crystalline phase, as indicated by observed peaks for the MSNPCLCR spectrum. XRD data show that the peaks of loaded-MSNs were shifted and had lower intensity compared to functionalized-MSNs. Probably this is connected with an increase of thickness of MSNs’s pore’s walls following filling of the pores with prodrugs. These results demonstrate that most of both agents will be trapped in MSNP in the non-crystalline phase, which can offer benefit in the enhancement of solubility [52]. Indeed, the solubility of crystal drugs is lower than that of non-crystalline/amorphous drugs [53].

FTIR characterization confirmed the presence of dual CL and CR natural agents and also the polymeric coating by revealing several characteristic corresponding peaks for the nanoformulations. This observation is inconsistent with previous results for two drugs combined in MSNs, such as the dual loading of the anticancer drug pemetrexed and the natural agent ellagic acid [54]. One important characterization is zeta potential measurement. The zeta potential results indicated that the nanoformulations suspended in deionized water changed depending on pH level from acidic to neutral to alkaline. The MSNPCLCR/CSCEFA nanoformulation seems the most suitable because it had a positive zeta potential of 43.3 ± 0.7, 25.6 ± 1.2, and 0.7 ± 0.6 at respective pH values of 2.5, 5, and 7 (from acidic to neutral). These results agree with our earlier zeta potential results for CL-loaded MSNs coated with chitosan-amino acid–conjugated FA [11]. This feature is more likely to induce cellular uptake of the nanoformulation into cells by interaction with negative charges of the cancer cell membrane [10,55,56]. The results suggest the importance of the fabricated nanoformulation having a polymeric coating to generate a higher cellular uptake in cancer cells.

The anticancer evaluations demonstrate that the nanoformulations significantly inhibited the proliferation of MCF-7, HCT-116, HOS, and A549 cell lines more so than did free CL and CR. The nanoformulations containing dual agents (MSNPCLCR and MSNPCLCR/CCFA) were more effective than MSNPCL (i.e., CL only). The results are in line with published data for the dual delivery system of anticancer therapies encapsulated in MSNs compared with free components [45,47,54,57,58]. The IC50 confirmed that both nanoformulations with dual agents induced selective killing of HCT-116 colon cancer cells, followed by A549, HOS, and MCF-7. These results indicate the superiority of these dual nanoformulations in selectively targeting colon cancer cells, with minimal cytotoxicity to normal healthy cells.

Apoptosis is part of different physiological and pathological responses [59], and the antitumor effects of most anticancer drugs usually relate to apoptosis [60,61]. One important step in the apoptosis pathway is caspase-3 activation, which can lead to cleaving and inactivation of many proteins in cancer cells [62]. Caspase-3 promotion is crucial for constraining cancer cell proliferation [63,64,65], and here we found more upregulation of caspase-3 expression in cells treated with nanoformulations versus free CR or CL. 

Another important characteristic of the apoptosis pathway is p53 protein activation. The interaction of cell cycle inhibition and apoptosis is important for anticancer therapy, and p53 is crucial to tumor sensitivity to drugs [66,67]. The results showed that the fabrication nanoformulations were specially designed with targeted delivery of MSNPCLCR/CSCEFA nanoformulation activated p53 expression.

Bcl-2 inhibits programmed cell death in cancers and promotes cell survival, supporting cancer resistance to drugs [68,69]. Bax promotes cell death and strongly induces apoptosis in cancer cells presenting in inactivated states [61,70]. Therefore, modulating expression of both proteins (Bax upregulation, Bcl-2 downregulation) is crucial to generating apoptosis. Our results demonstrated that MSNPCLCR/CSCEFA was the most effective at modulating Bax (increasing) and Bcl-2 (decreasing) expression, by comparison with free natural agents. These results echo earlier findings with a co-delivery system fabricated by hollow MSNs with both doxorubicin and NVP-AEW 541 loaded inside, which elicited enhanced apoptosis through Bax activation and Bcl-2 inhibition in ovarian cancer stem-like cells [44].

## 5. Conclusions

We describe a new co-delivery system constructed by combining CR and CL without polymeric coating (MSNPCLCR, a core nanoformulation) and with a CS-CE-FA polymeric coating (MSNPCLCR/CSCEFA, a core-shell nanoformulation) using MSN as drug nano-vehicles. The dual delivery nanoformulation of both core and core-shell composed of ~18.1 wt.% (CL and CR) exhibited superior anticancer effects through enhanced apoptosis induction compared with single delivery of CL with 10.9 wt.% and free natural agents tested in equivalent concentrations.

Through in vitro tests with four cell lines—MCF-7 breast adenocarcinoma, HCT-116 colon cancer, HOS bone cancer, and A-549 non–small cell lung cancer—IC50 results showed that the dual delivery of MSNPCLCR and MSNPCLCR/CSCEFA was most efficient, in this order: HCT-116 > A549 > HOS > MCF-7. The lowest IC50 value was obtained for the coated nanoformulation (4.1 ± 0.05 µg/mL). Apoptosis in cancer cells improved following the dual delivery nanoformulations, with promoting expression of p53, caspase-3, and Bax and inhibiting expression of Bcl-2.

The promising findings from our study, using a co-delivery system for two natural agents with MSNs, should be verified through in vivo experiments to confirm the potential therapeutic anticancer effect.

## Figures and Tables

**Table 1:** Conditions for preparation of single and co-delivery nanoformulations using colchicine and curcumin natural agents and their physicochemical characteristics.

Formula	Preparation Conditions			Weight Loss wt.% ^a^ Total Prodrug Content (TPC wt.%)	S_BET_ (m^2^/g) ^b^	Total Pore Volume ^c^ (CC/g)
	Drug: Nanoparticles Ratio	Volume/Solvent	Temperature/ Stirring Speed			
MSNP				21.5	206.2	0.689
F1: MSNPCL	1:3	10 mL CL/d. water	RT (24 h)/270 rpm	32.4 TPC ~ 10.9	144.3	0.528
F2: MSNPCLCR	300 mg MSNPCL/ethanol containing 100 mg CR RT (24 h)/250 rpm			50.5 TPC ~ 18.1	15.1	0.165
F3: MSNPCLCR/CSCEFA	MSNPCLCR was resuspended in CS-CE-FA/ RT (24 h)/250 rpm			60.8 TPC ~ 18.1 10.3 (as shell coating)	1.0	0.188

^a^ Data were obtained from thermogravimetric analysis. ^b^ Specific surface area measured from BET measurement. ^c^ Total pore volume at ~0.989 P/Po. RT, room temperature. The TPC content was calculated based on the weight loss values wt.% as follows: CL wt.% in MSNPCL = MSNPCL − MSNP × 100; CL and CR wt.% in MSNPCLCR = MSNPCLCR − MSNPCL × 100; and CL and CR in MSNPCLCR/CSCEFA calculated as 18.1 wt.%.

**Table 2:** IC50 (µg/mL) of free natural agents and nanoformulations against different cancer cell lines.

Treatments	Cancer Cell Line			
	MCF-7	HCT-116	HOS	A549
MSNPCL	65.3 ± 0.05	4.8 ± 0.05	68.7 ± 0.06	17.1 ± 0.05
MSNPCLCR	23.7 ± 0.05	6.2 ± 0.05	18.6 ± 0.05	16.6 ± 0.05
MSNPCLCR/CSCEFA	20.2 ± 0.06	4.1 ± 0.05	12.0 ± 0.05	16.6 ± 0.06
CL	101.3 ± 0.14	92.6 ± 0.15	111.0 ± 0.08	89.0 ± 0.07
CR	98.8 ± 0.13	116.7 ± 0.16	47.9 ± 0.05	38.6 ± 0.06

IC50 concentration was calculated for nanoformulations based on the content of CL and/or CR in the nanoformulations.

## Data Availability

Not applicable.
